# Beyond the Bot: A Dual-Phase Framework for Evaluating AI Chatbot Simulations in Nursing Education

**DOI:** 10.3390/nursrep15080280

**Published:** 2025-07-31

**Authors:** Phillip Olla, Nadine Wodwaski, Taylor Long

**Affiliations:** 1 College of Health Professional, University of Detroit Mercy, Detroit, MI 48221, USA; 2 McAuley School of Nursing, University of Detroit Mercy, Detroit, MI 48221, USA; wodwasnk@udmercy.edu (N.W.); brazeltk@udmercy.edu (T.L.)

**Keywords:** AI chatbots, simulation-based learning, AI chatbot evaluation framework, AIMS evaluation framework

## Abstract

**Background/Objectives:** The integration of AI chatbots in nursing education, particularly in simulation-based learning, is advancing rapidly. However, there is a lack of structured evaluation models, especially to assess AI-generated simulations. This article introduces the AI-Integrated Method for Simulation (AIMS) evaluation framework, a dual-phase evaluation framework adapted from the FAITA model, designed to evaluate both prompt design and chatbot performance in the context of nursing education. **Methods:** This simulation-based study explored the application of an AI chatbot in an emergency planning course. The AIMS framework was developed and applied, consisting of six prompt-level domains (Phase 1) and eight performance criteria (Phase 2). These domains were selected based on current best practices in instructional design, simulation fidelity, and emerging AI evaluation literature. To assess the chatbots educational utility, the study employed a scoring rubric for each phase and incorporated a structured feedback loop to refine both prompt design and chatbox interaction. To demonstrate the framework’s practical application, the researchers configured an AI tool referred to in this study as “Eval-Bot v1”, built using OpenAI’s GPT-4.0, to apply Phase 1 scoring criteria to a real simulation prompt. Insights from this analysis were then used to anticipate Phase 2 performance and identify areas for improvement. Participants (three individuals)—all experienced healthcare educators and advanced practice nurses with expertise in clinical decision-making and simulation-based teaching—reviewed the prompt and Eval-Bot’s score to triangulate findings. **Results:** Simulated evaluations revealed clear strengths in the prompt alignment with course objectives and its capacity to foster interactive learning. Participants noted that the AI chatbot supported engagement and maintained appropriate pacing, particularly in scenarios involving emergency planning decision-making. However, challenges emerged in areas related to personalization and inclusivity. While the chatbot responded consistently to general queries, it struggled to adapt tone, complexity and content to reflect diverse learner needs or cultural nuances. To support replication and refinement, a sample scoring rubric and simulation prompt template are provided. When evaluated using the Eval-Bot tool, moderate concerns were flagged regarding safety prompts and inclusive language, particularly in how the chatbot navigated sensitive decision points. These gaps were linked to predicted performance issues in Phase 2 domains such as dialog control, equity, and user reassurance. Based on these findings, revised prompt strategies were developed to improve contextual sensitivity, promote inclusivity, and strengthen ethical guidance within chatbot-led simulations. **Conclusions:** The AIMS evaluation framework provides a practical and replicable approach for evaluating the use of AI chatbots in simulation-based education. By offering structured criteria for both prompt design and chatbot performance, the model supports instructional designers, simulation specialists, and developers in identifying areas of strength and improvement. The findings underscore the importance of intentional design, safety monitoring, and inclusive language when integrating AI into nursing and health education. As AI tools become more embedded in learning environments, this framework offers a thoughtful starting point for ensuring they are applied ethically, effectively, and with learner diversity in mind.

## 1. Introduction

Simulation-based education (SBE) is a cornerstone of nursing education, offering learners structured opportunities to build clinical reasoning, technical proficiency, and interpersonal skills in a risk-free environment. In particular, the Healthcare Simulation Standards of Best Practice^®^ (HSSOBP), developed by the International Nursing Association for Clinical Simulation and Learning (INACSL), have played a critical role in shaping the design, facilitation, and evaluation of simulation activities across academic and clinical settings [1,2]. These standards underscore the importance of evidence-based practice, psychological safety, scenario fidelity, structured debriefing, and learner-centered assessment—ensuring that simulation aligns with curricular goals and ultimately supports safe, high-quality patient care. In parallel, the Association for Simulated Practice in Healthcare (ASPiH) provides similar guidance in the United Kingdom and Europe, with a shared focus on scenario design, faculty development, and quality assurance [3]. Together, these frameworks form the pedagogical and ethical foundation upon which effective nursing simulations are built, supporting student competence, clinical judgment, and confidence before entering complex practice environments [1,2,3,4,5].

Simulation is more than a teaching tool—it is a vital bridge between theoretical instruction and real-world applications. The American Association of Colleges of Nursing (AACN) underscores this in its 2021 Essentials, identifying simulation-based learning as essential to the development of multiple domains, including Domain 2 (Person-Centered Care), Domain 4 (Scholarship for the Nursing Discipline), and Domain 9 (Quality and Safety).

Through immersive practice, nursing students cultivate critical thinking, clinical judgment, and ethical decision-making skills without placing actual patients at risk. Simulation also fosters reflective learning, enabling learners to make mistakes, receive feedback, and improve—principles central to competency-based education. The Clinical Judgment Model (CJM), often integrated into simulation, further enhances learners’ ability to synthesize data, reason logically, and respond compassionately. As artificial intelligence (AI) tools such as chatbots begin to augment simulation scenarios, educators must ensure these innovations maintain the fidelity, learner engagement, and accountability defined in national standards. The rise in AI chatbots and conversational agents presents both opportunities and complexities for healthcare education. AI-powered tools, especially those utilizing large language models, provide scalable, adaptive solutions for clinical learning, patient interaction, and real-time formative assessment in nursing However, as these technologies integrate into simulation-based learning, questions arise regarding their accuracy, safety, ethical use, and educational validity. Existing simulation frameworks, while robust, are not fully designed to assess the nuanced behaviors and outputs of AI systems. Manual evaluation processes, though foundational, are increasingly strained by the speed and scale of AI content generation [5].

The effectiveness of AI chatbots in healthcare education depends not just on user-facing design but on deep integration with backend clinical data and real-time analytics. As shown during the COVID-19 pandemic, chatbots embedded within large digital health ecosystems like the WHO’s Facebook chatbot can enhance crisis communication and adaptability. This demonstrates how health crisis communication can be significantly improved when chatbots are embedded in larger digital health ecosystems that combine artificial intelligence with global-scale data repositories and knowledge graphs [6]. Future nursing education tools should adopt similar models, linking chatbots with institutional guidelines, EHRs, and public health databases to ensure responses are accurate, personalized, and contextually relevant. Chatbots can simulate clinical conversations, guide student decision-making, and reinforce safety protocols beyond traditional classroom settings [7].

To guide educators in evaluating both the design and performance of AI-enhanced simulation tools, this study introduces the AI-Integrated Method for Simulation Evaluation (AIMS) Framework—a dual-phase, iterative model grounded in best practices from healthcare simulation and AI assessment literature. As AI becomes more embedded in educational settings, a number of evaluation frameworks have emerged to guide responsible implementation. One leading example is the Framework for AI Tool Assessment in Mental Health (FAITA), a domain-based rubric developed to evaluate AI-powered tools in mental health settings [8]. FAITA introduces key domains such as clinical credibility, user experience, health equity, user agency, transparency, and crisis management [8]. This practical scoring system equips educators, developers, and institutions with tools to assess cultural sensitivity, clinical integrity, and emotional safety. Although designed for mental health, FAITA’s person-centered, ethical approach makes it highly adaptable to broader educational settings, including nursing simulation [8,9]. Complementary to FAITA, recent scholarly reviews have synthesized a growing body of evidence focused on evaluating chatbots and conversational agents in healthcare [8]. These reviews advocate for the adoption of multi-stage evaluation frameworks that align with the World Health Organization (WHO) guidelines and other globally recognized digital health evaluation strategies [10,11,12,13]. Common elements within these models include feasibility, usability, clinical effectiveness, real-world implementation, and sustainability. Key evaluation categories span functionality, safety and information quality, user experience, health outcomes, and cost-effectiveness, while also considering diverse user characteristics critical to successful implementation. Importantly, these frameworks move beyond assessing technical capabilities alone, highlighting the need to measure equity, patient engagement, and ethical dimensions of chatbot use. As AI tools increasingly mediate patient education and simulation, comprehensive evaluation models become essential to ensure that innovation does not come at the expense of inclusivity, accuracy, or long-term impact on care outcomes [12,13,14]. Innovative AI-powered validation approaches are also gaining traction in healthcare simulation. One such example is the “3-bot” method, which employs three AI agents to simulate interaction between a patient, provider, and evaluator [15]. These agents work collaboratively to generate, interpret, and critique dialog based on predefined evaluation criteria. This method allows for scalable, safe, and efficient testing of chatbot responses, significantly reducing reliance on human evaluators while preserving analytical depth and consistency. Despite its promise, this model underscores the ongoing need for broader, more context-sensitive evaluation frameworks—particularly as chatbots are applied in ethically complex or emotionally charged clinical environments. As healthcare simulation embraces AI-driven tools, maintaining fidelity, safety, and cultural humility must remain at the forefront of all evaluative efforts [16]. In summary, simulation continues to serve as a foundational pillar in nursing education, supporting the development of competent, reflective, and ethically grounded practitioners. As AI-driven tools enter the simulation space, a hybrid evaluative approach is needed—one that marries the pedagogical strengths of frameworks like INACSL’s HSSOBP and ASPiH’s standards with the technological specificity of models like FAITA and the 3-bot method. To support this evolution, the AI-Integrated Method for Simulation Evaluation (AIMS) evaluation framework provides a dual-phase, iterative structure tailored specifically for simulation-based education. For nurse educators, the goal is not only to embrace innovation but to ensure that AI-enhanced simulations remain learner-centered, inclusive, and aligned with the core principles of nursing: safety, integrity, and compassionate care.

## 2. Materials and Methods

### 2.1. Framework Design

The AI-Integrated Method for Simulation (AIMS) Evaluation Framework was developed to address a critical gap in the structured assessment of AI chatbots for nursing simulation-based education. The framework was grounded in an extensive review of peer-reviewed literature, national simulation standards, and global guidance on digital health evaluation. Foundational elements were drawn from FAITA model, and expanded to align with curren best practices in simulation fidelity standards, pedagogical priorities, and emerging AI ethics in healthcare education. The AIMS Framework’s was designed as a Dual-Phase Evaluation Model. Phase 1 focuses on the instructional prompt design, ensuring the upstream factors such as educational alignment, psychological safety, inclusivity, and ethical clarity are robustly addressed. Phase 2 evaluates the chatbot’s real-time performance, examining its output for clinical accuracy, cultural responsiveness, dialog flow, feedback quality, and user agency. The domains in both phases were selected through an iterative concept-mapping process that linked best practice guidelines from established simulation standards, the WHO digital health evaluation toolkit, and recent literature on AI-assisted simulation.

To operationalize this framework, the research team built both the Emergency Response Simulation Bot and the prototype Eval-Bot using OpenAI’s GPT-4.0 large language model. This specific choice was guided by GPT-4.0’s advanced natural language processing capabilities, flexibility for prompt engineering, and its ability to generate realistic, contextually appropriate dialog aligned with nursing education scenarios. The Simulation Bot’s scenario flow, branching logic, and feedback scripts were developed through iterative prompt testing and fine-tuning within the GPT-4.0 API to maintain clinical relevance and learner engagement. The simulation bot could be interacted with using both text and voice modalities, but interaction with text was the recommended approach.

**Figure 1:**
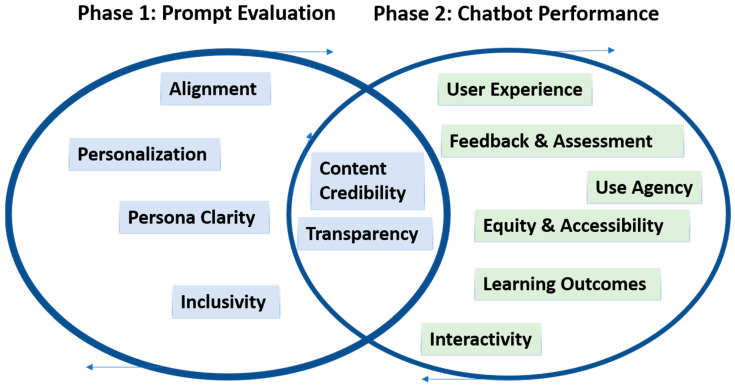
AIMS evaluation framework.

The final framework is visually represented in Figure 1, which outlines the abstract architecture of AIMS, including its key modules (Prompt Evaluation, Chatbot Performance Assessment) and core functions (rubric scoring, structured feedback, iterative refinement). This structured yet adaptable design ensures that the AIMS Framework can guide simulation specialists, educators, and developers in evaluating and improving AI-enhanced educational tools while preserving the integrity and learner-centered values of simulation-based pedagogy.

Rubric domains for both phases were developed through concept mapping and refined through internal expert review by simulation faculty to ensure that the framework could guide both formative evaluation and ongoing improvement. The final framework is visually represented in Figure 1, which outlines the abstract architecture of AIMS, including its key modules (Prompt Evaluation, Chatbot Performance Assessment) and core functions (rubric scoring structured feedback, and iterative refinement). This structured yet adaptable design ensures that the AIMS Framework can support simulation specialists, educators, and developers in learner-centered values of simulation pedagogy. Table 1 and Table 2 summarize the domains assessed in both Phase 1 and Phase 2, respectively.

### 2.2. Demonstration Example

While Phase 1 of the AIMS Framework was successfully implemented to evaluate the instructional quality of a chatbot prompt, the full deployment of Phase 2—measuring chatbot performance in an active simulation—requires future empirical research. This would include structured observations, pre- and post-simulation assessments, and learner surveys designed to capture cognitive, affective, and behavioral outcomes. Such a multi-dimensional validation approach is essential to fully establish the educational effectiveness of AI-enhanced simulations. However, this article focuses on framework development and Phase 1 testing; Phase 2 validation will be pursued in future studies.

To illustrate the application of Phase 1, an educational simulation currently used in undergraduate healthcare education was retrospectively evaluated using the AIMS Framework. The scenario involves an Emergency Response Simulation bot designed by the research team using Open AI’s GPT-4.0 large language model to guide nursing and healthcare students through a simulated hospital emergency during a power outage. The chatbot prompts learners to select a professional role, navigate escalating clinical decisions, and receive feedback based on their actions and judgment.

The Emergency Response Simulation Bot and the prototype Eval-Bot were both built using OpenAI’s GPT-4.0 large language model. The simulation bot’s scenario design, role-based branching, and feedback scripts were created through iterative prompt engineering with the GPT-4.0 API to ensure accuracy and realistic pacing. Eval-Bot was developed using the same underlying model but specifically fine-tuned to align with the AIMS Framework Dual-Phase Evaluation rubric. This setup enabled consistent scoring, structured feedback, and rapid refinements to prototype educational chatbots. These implementation details are provided to support other educators and researchers in replicating or adapting similar AI-enhanced simulations.

To ensure the trustworthiness of this initial Phase 1 evaluation, a small panel of three independent human evaluators also reviewed the chatbot prompt and the scores generated by Eval-Bot. These evaluators were selected based on their professional background as registered nurses and nursing faculty, each with at least five years of clinical teaching, simulation design, or curriculum assessment experience in undergraduate nursing education. All hold advanced nursing degrees and prior training in simulation-based learning or digital pedagogy. Evaluators were not involved in the original chatbot design, helping to reduce bias, and did not have access to each other’s scores during their review. Although they were not blinded to Eval-Bot’s rubric ratings, they provided independent numeric scores and narrative feedback to triangulate the automated evaluation. This retrospective evaluation of the chatbot prompt was considered minimal risk and did not involve student participants or identifiable data. Therefore, the activity did not constitute human subjects’ research under federal guidelines and was deemed exempt by the university’s Institutional Review Board. The professional evaluators voluntarily agreed to contribute their expertise as faculty reviewers to support framework development. This blended approach allowed the team to compare human judgment with algorithmic scoring, identify areas of alignment and discrepancy, and refine the AIMS Framework rubric for future validation.

The following prompt, shown in Table 3, was used in the chatbot builder to create a link that was deployed to the students to access the chatbot. The prompt has been extracted from the chatbot and used in this article.

To support scalable and consistent evaluations, the researchers developed an AI-powered chatbot named Eval-Bot, trained specifically on the AIMS Framework Dual-Phase Evaluation rubric. Eval-Bot was designed to autonomously assess chatbot prompts and simulate learner interactions using the established rubric criteria from Phase 1 and Phase 2. By leveraging a large language model, fine-tuned to the AIMS Framework parameters Eval-Bot generated structured feedback, quantitative scores and narrative rationale for each domain. This tool enabled rapid prototyping and iterative refinement of educational chatbots using consistent evaluative standards. Additional details on prompt architecture, technical designs, and Eval-Bot outputs are included in the Supplementary Materials Files S1–S3.

## 3. Results

To assess the instructional quality of the original prompt, Phase 1 of the AIMS Framework was applied using an AI-based tool (Eval-Bot). Each evaluator independently scored the prompts across six domains of Phase 1 using a 0–2 scoring scale. In this system higher scores reflected stronger alignment with the AIMS Framework principles, including education coherence, psychological safety, and inclusivity.

The resulting scores and narrative rationales were synthesized and presented in Table 4 to identify key areas of agreement and divergence between the human and AI evaluations. This dual-method approach illustrates the potential for AI-assisted evaluation to complement human judgment, promote consistency, and accelerate feedback cycles in the iterative design of AI-enhanced simulations.

Both evaluators, human and AI based (Eval-Bot) identified strengths in the original prompt alignment with learning objectives and role clarity. These strengths underscore the instructional utility of the prompts foundational structure. However, the evaluation also revealed challenges related to personalization and cultural sensitivity, highlighting areas where the prompt did not fully adapt Content complexity to meet diverse learner needs. This highlights an important trade-off: in time-sensitive scenarios like emergency planning, frontline nurses may have limited capacity to personalize chatbot interactions, see Table 5. This suggests that AI tools must be designed as supplemental aids rather than replacements for real-time human clinical judgment, with ongoing research needed to strengthen their cultural and contextual adaptability. Consistent weaknesses emerged in inclusivity, transparency, and evidence-based grounding, with the human evaluators specifically noting the lack of explicit cues for cultural sensitivity and limited adaptability to learner diversity. This highlights the following important distinction: while Eval-Bot demonstrated competence in recognizing structural coherence, human reviewers were more attuned to nuanced pedagogical and ethical elements. This divergence illustrates the complementary value of using both human and AI perspectives in Phase 1 of the AIMS Framework, supporting an iterative feedback loop that aligns with evolving pedagogical and clinical priorities.

The dual-phase approach of the AIMS Framework highlights a critical reality that flawed prompt design can lead to inaccurate, biased, or unsafe chatbot interactions, which can have real-world consequences in nursing education and clinical preparation. By rigorously evaluating prompts upstream, educators can reduce the risk of downstream AI miscommunication. The embedded feedback loop further supports iterative refinement, ensuring that both prompt and chatbot performance can adapt as pedagogical needs, clinical guidelines, and learner diversity evolve. This phased safeguard is especially important in healthcare contexts, where flawed AI advice could influence critical thinking in high-stakes, time-sensitive settings such as emergency planning.

The primary contribution of this work is the development and demonstration of the AIMS Framework and prototype Eval-Bot, which together offer a practical, dual-phase method for systematically evaluating the design and performance of AI-powered chatbots in nursing simulation. However, the real-time demands of emergency response highlight a trade-off: while chatbots can enhance preparedness and practice, front-line nurses may have limited capacity to consult them during actual crises. This underscores the need for integrated, blended learning that builds confidence in decision-making when time is constrained. To address these gaps, the prompt was revised to preserve instructional integrity while intentionally enhancing learner-centered features in alignment with the AACN Essentials, INACSL Healthcare Simulation Standards of Best Practice, and best practices in AI-augmented simulation design.

Phase 2 implications based on Phase 1 findings.

The AIMS Framework emphasizes the interconnected nature of prompt quality (Phase 1) and chatbot performance (Phase 2). Based on Phase 1 results, the following Phase 2 challenges were anticipated:

- Content Accuracy: The absence of references to evidence-based guidelines risks reducing the factual precision of chatbot responses.
- User Trust and Accessibility: A lack of transparency regarding AI limitations and insufficient inclusive language may reduce user trust and engagement, particularly among diverse learners.
- Feedback Consistency: Without predefined expectations or a formative rubric, the chatbot may deliver feedback inconsistently, diminishing its educational value.

Looking forward, the AIMS Framework could evolve to include backend interoperability with large-scale clinical knowledge networks, electronic health records, and global medical libraries to ensure responses remain current, cross-verified, and culturally responsive. Such connected systems, similar to the WHO’s pandemic-era chatbots, could further reduce the risk of isolated or outdated AI advice.

In the interim, however, prompt-level refinements remain critical to safeguarding instructional integrity and learner trust as AI tools improve. This revised prompt was intentionally crafted to perserve the instructional integrity of the simulation while enhancing learner-centered features aligned with the AACN Essentials, INACSL Healthcare Simulation Standards of Best Practice, and the guiding principles of the AIMS Framework. It reflects best practices in AI-augmented healthcare education by prompting inclusivity, transparency, ethical guidance, and evidence-based dialog within the simulation experience.

## 4. Discussion

This study introduces the AI-Integrated Method for Simulation Evaluation (AIMS) Framework, a novel Dual-Phase Evaluation Model to assess the instructional design and performance of AI-powered chatbots within educational simulations. Adapted from FAITA and guided by the extensive literature review, AIMS addresses a critical gap between established simulations and the complexities introduced by generative AI technologies. Its application to a real-world chatbot scenario in an emergency planning course demonstrated the frameworks flexibility, relevance, and practical utility for simulation specialists, instructional designers, and healthcare educators.

Phase 1 of the evaluation revealed notable strengths in educational alignment and clarity of purpose, confirming that the original prompt was effective in supporting clinical reasoning and role-specific decision-making. However, moderate shortcomings in safety signaling and cultural inclusivity were also identified. These results align with growing concerns in AI literature, where large language models—unless explicitly guided—can reinforce dominant cultural narratives or fail to accommodate diverse learner perspectives [7,10]. For example, although the original simulation facilitated progressive scenario development, it lacked personalized adjustments based on learner background or skill level, which could diminish psychological safety and user trust.

The integration of Eval-Bot, an autonomous AI evaluator trained using the AIMS Framework, further underscores the potential for scalable evaluation in simulation design. Similarly to recent innovations like the “3-bot” model, which uses AI agents to simulate clinical interactions and critique responses in real time [15], Eval-Bot provided reliable and structured feedback consistent with human judgment. Its most significant contribution lay in its capacity to deliver repeatable, domain-specific evaluations—an essential function as institutions scale the use of AI chatbots in education and training.

Importantly, the study reinforces the prompt engineering is a foundational design task, not merely a technical step. The structure and wording of a prompt shape the chatbot’s reasoning, tone, and user engagement. Revisions guided by Phase 1 findings—focused on transparency, inclusivity, and role personalization—were projected to improve multiple Phase 2 outcomes, including content accuracy, learner agency, and emotional safety. This supports a growing body of evidence that intentional prompt design significantly enhances chatbot performance and educational value [8,9]. Furthermore, the AIMS Framework reflects core principles of simulation-based education, including formative assessment, reflective practice, and iterative improvement. By identifying gaps at the prompt level before full deployment, educators can prevent harm and optimize learning experiences. This mirrors longstanding simulation pedagogy emphasized by the INACSL Healthcare Simulation Standards of Best Practice^®^ and ASPiH’s quality guidelines, which prioritize learner safety, fidelity, and intentional design [1,2,3,4,5].

The real-time demands of emergency response scenarios also highlight an important trade-off while AI chatbots’ can enhance preparedness and foster critical thinking, front-line nurses may have limited capacity to rely on these tools during high-pressure situations. This underscores the need to position chatbots as supplementary learning aids that build decision-making confidence before crises occur, rather than as replacements for clinical judgment in urgent settings.

Looking forward, the findings implicitly argue for a more integrated approach to AI in healthcare education. As standalone chatbots—no matter how well-designed—remain limited without trusted data sources, future iterations of the AIMS Framework could evolve to incorporate backend interoperability with large-scale clinical knowledge networks, electronic health records, and global medical libraries. Such connections would enable real-time cross-domain verification, improving clinical accuracy and cultural responsiveness. Embedding these standards for connectivity and ethical oversight into industry guidelines would help ensure that AI-driven simulations align with the highest standards of patient safety and inclusivity.

Taken together, the AIMS Framework offers more than a static rubric—it provides aniterative ataptable pathway for simulation educators to responsibly integrate AI tools into nursing education. By balancing technological innovation with pedagogical rigor and ethical safeguards, this framework lays the groundwork for a future where AI-augmented learning environments are not only technically robust but also human-centered and responsive to the evolving needs of learners and the communities they serve.

## 5. Limitations

While this initial demonstration of the AIMS Framework highlights its promise for guiding AI evaluation in nursing simulation, several important limitations should be acknowledged. This study focused on a single, scenario-specific use case—an Emergency Response Simulation Bot—which naturally narrows the scope of what can be generalized to other educational contexts, clinical specialties, or diverse learner populations. Although the scenario was chosen for its relevance to undergraduate nursing education, future applications should test the framework with varied topics, learner groups, and simulation complexities.

Another limitation involves the human evaluators who reviewed the chatbot prompt alongside the AI-based Eval-Bot. The panel consisted of three experienced nursing faculty with backgrounds in clinical teaching, simulation design, and curriculum evaluation. While they brought valuable insight, their perspectives cannot fully capture the range of pedagogical approaches or cultural contexts that exist across institutions and countries. In addition, the evaluators were not blinded to Eval-Bot’s rubric scores during their review. While each provided independent ratings and narrative feedback to help triangulate findings, the lack of blinding does introduce the possibility of confirmation bias.

This demonstration also relied on retrospective evaluation. As such, the faculty evaluators participated voluntarily and no student data were collected, meaning that the project qualified for IRB exemption. Nonetheless, future studies that include direct student engagement or new data collection should follow full human subjects research protocols, including informed consent.

A further consideration is that this paper reports only on the Phase 1 application of the AIMS Framework—which focuses on prompt design quality—while Phase 2, which examines real-world chatbot performance, learner outcomes, and behavioral changes, remains to be tested. Robust Phase 2 studies, including structured observations, learner feedback, and pre/post assessments, are needed to fully validate how well AI chatbots align with simulation learning goals over time.

Finally, the chatbots described here, including Eval-Bot, were developed using OpenAI’s GPT-4.0 large language model. Although this choice allowed rapid iteration and detailed feedback, other language models may perform differently. Researchers and educators seeking to replicate or adapt this approach should be mindful of possible variation in how different AI tools interpret prompts, handle inclusivity, or generate scenario dialog.

Taken together, these limitations point to the need for future work that includes broader institutional partnerships, larger and more diverse expert panels, multi-language scenarios, and a more rigorous assessment of Phase 2 performance. By addressing these areas, the AIMS Framework can continue to evolve as a practical, trustworthy guide for integrating AI chatbots into nursing and healthcare simulation—always grounded in the principles of safety, ethical practice, and caring pedagogy.

## 6. Conclusions

This article introduces the AIMS Framework, a dual-phase evaluation framework designed to assess the quality and impact of AI chatbots in educational simulation contexts. By explicitly separating the evaluation of the prompt (Phase 1) from the evaluation of the chatbot’s performance (Phase 2), this model offers a novel, replicable structure for ensuring the pedagogical integrity, safety, and effectiveness of AI-driven learning tools.

The application of the dual-phase framework to an emergency response simulation prompt provided valuable insight into the relationship between prompt design and chatbot performance. While the original prompt displayed strong alignment with educational goals and clearly defined roles, it lacked sufficient attention to inclusivity, transparency, and learner personalization. Phase 1 findings, derived from both human and AI evaluators, projected potential Phase 2 challenges in areas such as dialog consistency, learner engagement, and equitable representation, underscoring the framework’s diagnostic utility.

Unlike existing models such as FAITA, the WHO-aligned digital health evaluation stages, or the 3-bot validation methodology, the AIMS evaluation framework introduces a novel emphasis on prompt engineering as a foundational element in AI chatbot development. This focus addresses a critical gap in healthcare education literature, where the quality of initial inputs often determines the trajectory of learner interaction, safety, and perceived authenticity.

By integrating simulation-based education principles with AI-specific assessment criteria, the AIMS framework offers a practical and forward-thinking strategy for evaluating and refining AI-enhanced simulation tools. It also acknowledges the real-world trade-offs of chatbots as supplemental aids, not replacements for urgent decision-making on the front line, highlighting the need for blended learning approaches that build clinician confidence when time is limited

Looking ahead, the framework’s phased design could be expanded to include backend connectivity with large-scale clinical knowledge networks, EHR systems, or global medical libraries to strengthen cross-domain verification and real-time accuracy.

It not only supports educators and simulation specialists in making informed design decisions but also advances responsible innovation by centering educational alignment, safety, and learner inclusivity. Embedding industry standards for interoperability, ethical oversight, and cross-domain validation will be vital as these tools evolve within healthcare education.

As AI technologies become more prevalent in academic settings, the AIMS evaluation framework equips institutions with a rigorous yet adaptable model to uphold the values of ethical, learner-centered education—while remaining flexible enough to evolve alongside technological and pedagogical advancements

## Figures and Tables

**Table 1:** Promt Evaluation Domains.

Domain	Description
**Clinical/Content Credibility**	Determines whether the prompt instructs the chatbot to use trusted, evidence-based sources.
**Alignment**	Combines both educational and cognitive alignment to evaluate clarity of objectives, instructional level, and depth of engagement.
**Persona Clarity**	Assesses whether the prompt adequately defines the chatbot’s role, tone, and identity (e.g., tutor, coach, assistant).
**Inclusivity and Cultural Competence**	Verifies the prompt encourages culturally respectful and accessible communication.
**Transparency**	Confirms that the prompt requires the chatbot to disclose its AI identity and limitations.
**Personalization**	Assesses if the prompt allows for adaptable responses based on learner profiles or needs.

**Table 2:** Chatbot Performance Domains.

Domain	Description
**Credibility and Accuracy**	Measures whether the chatbot delivers correct, evidence-based, and current educational information.
**User Experience**	Evaluates the interface’s ease of use, conversational flow, clarity, and learner engagement.
**Feedback or Assessment**	Assesses the chatbot’s ability to give timely, clear, and constructive educational feedback.
**User Agency**	Considers whether learners can guide, pause, or exit the interaction as desired.
**Equity and Accessibility**	Reviews whether the chatbot serves diverse learners, including those with disabilities or low literacy.
**Transparency**	Checks if the chatbot clearly identifies as non-human and explains its capabilities and limitations.
**Learning Outcomes**	Determines whether users demonstrate knowledge or skill acquisition following chatbot interaction.
**Interactivity Quality**	Evaluates how well the chatbot maintains coherent, responsive, and context-aware dialog.

**Table 3:** Prompt structure for emergency planning simulation.

Heading	Details
**Original Prompt**	You are an Emergency Response Simulation Bot for healthcare students. Your purpose is to guide individual learners through a real-time simulation involving a sudden power outage in a hospital Emergency Room. You act as both the scenario engine and feedback coach. Students will choose a professional role and respond to crisis prompts. You will escalate the scenario in stages, ask them to make decisions, and provide contextually appropriate responses. At the end of the simulation, you will provide feedback based on their actions.
**Student Instructions**	**Displayed on Start:** Welcome to the Metro General Hospital Emergency Simulation. A major thunderstorm has caused a total power outage in the Emergency Room. Please begin by telling me your selected role: ER Charge Nurse, Facilities Coordinator, Informatics Lead, Communications Officer, or On-call Physician. I will guide you through the incident. Once you’re finished, type: “End simulation and provide feedback.”
**Role Activation**	Once the student provides a role, adapt all events and tasks based on that role. Use realistic medical terminology and time-sensitive updates. Keep interactions concise but convey urgency.
**Scenario Phases**	**Phase 1:** Report the basic situation (total power loss; generator not activating). **Phase 2–3:** Introduce 2–3 incident developments (e.g., patient arrival, equipment failure, staff panic, communication outage). After each escalation, prompt: “What do you do next?”
**Incident Escalations**	A trauma patient arrives by ambulance needing immediate intervention. Battery-powered ventilators are failing. Backup generator clicks in but only partially restores power. Staff are unsure of next steps, requesting direction. A local TV news crew calls for a statement.
**Acceptable Student Prompts**	Example responses may include: “Send emergency message to staff.” “Assess ventilator-dependent patients.” “Switch to paper charting.” “Contact facilities to investigate generator room.” “Reroute incoming patients to another hospital.”
**Feedback Generation**	When the student types **End simulation and provide feedback**, deliver a structured debrief: What they handled well Critical issues they missed How they could improve coordination or prioritization A 3–5-bullet summary or short paragraph with positive reinforcement and improvement tips
**Example Interaction Flow**	**Student:** I am the ER Charge Nurse **AI:** Power has failed across the hospital. Emergency lights are on. The generator has not kicked in. Three ventilator-dependent patients are critical. What’s your first action? … **Student:** End simulation and provide feedback **AI:** Thank you. Here’s your feedback based on the choices you made: You demonstrated good prioritization in stabilizing critical patients. Communication with staff was delayed, which could cause confusion. You correctly escalated to the facilities team. Consider developing a backup plan for electronic chart access earlier.

**Table 4:** Results from Phase 1 evaluation.

Domain	AI Score	AI Rationale	Human Score	Human Rationale
**Content Credibility**	1	Lacks specific reference to evidence-based practices or trusted protocols.	0	No mention of evidence-based standards to guide simulation scenarios.
**Alignment**	2	Clearly addresses decision-making and learning objectives.	2	Simulation scenarios are clear and logically structured.
**Persona Clarity**	2	Defines bot’s dual role as scenario engine and feedback coach.	2	Bot identity is established, though rubric-guided feedback may improve clarity.
**Inclusivity and Cultural Competence**	0	No indicators of inclusive language or cultural responsiveness.	0	No explicit safeguards to avoid bias or ensure cultural competence.
**Transparency**	1	Bot’s identity and limitations not clearly stated.	0	Omission of bot identity or exit options during simulation.
**Personalization**	1	Some personalization through role selection, but limited adaptability.	1	Role-based differentiation exists, but no adjustment to learning needs.
**Total Phase 1 Score**	7/12		5/12	

**Table 5:** Revised Prompt for emergency planning bot.

Section	Updated Prompt	Comments (AIMS Domains)
**Revised Prompt**	You are an Emergency Response Simulation Bot for healthcare students, built on evidence-based emergency management protocols (e.g., current AACN/Nursing Council guidelines). Your purpose is to guide individual learners through a real-time simulation of a sudden power outage in a hospital Emergency Room, modeling best practices for crisis response and patient safety. You act as both the scenario engine and feedback coach. Students will choose a professional role and respond to crisis prompts. You will escalate the scenario in stages, ask them to make decisions, and provide contextually appropriate responses. At the end of the simulation, you will provide feedback based on their actions.	**Credibility:** Cites AHA/Nursing Council protocols to ground scenario in evidence-based practice. **Transparency:** Maintains clear bot identity.
**Student Instructions**	**Displayed on Start:** “Welcome to the Metro General Hospital Emergency Simulation. A major thunderstorm has caused a total power outage in the Emergency Room. Please begin by telling me: (1) your professional role—ER Charge Nurse, Facilities Coordinator, Informatics Lead, Communications Officer, or On-call Physician—and (2) your level of experience in that role (novice, intermediate, expert). We will use clear, inclusive language throughout. Once you’re finished, type: **End simulation and provide feedback.**”	**Personalization:** Captures experience level to tailor scenario complexity. **Inclusivity:** Promises clear, inclusive language.
**Chatbot Behaviors**	**Role and Profile Activation:** Adapt events, tasks, and terminology to both the chosen role and reported experience level. Use realistic, inclusive medical terminology and time-sensitive updates. Keep interactions concise, urgent, and respectful of diverse communication styles. **Scenario Phases:** **Phase 1:** Report basic situation (total power loss; generator failure) aligned to standardized emergency checklists. **Phase 2–3:** Introduce 2–3 incident developments, pausing each time to ask, “What do you do next?” tailored to learner profile.	**Credibility:** Aligns Phase 1 with “standardized emergency checklists.” **Inclusivity:** Respects diverse communication styles. **Personalization:** Scenario pacing and language adapt to reported experience level.
**Incident Escalations**	Examples you can introduce as the scenario unfolds (following best-practice triage guidelines): A trauma patient arrives by ambulance needing immediate intervention. Battery-powered ventilators are failing. Backup generator clicks on but only partially restores power. Staff from diverse departments request direction, using clear role-based communication. A local TV news crew calls for a statement to be crafted in plain language.	**Alignment:** Tied to “best-practice triage guidelines.” **Inclusivity:** Includes clear role-based and plain-language communication for varied audiences.
**Acceptable Student Prompts**	Students may respond with actions such as: “Send an emergency message to staff via all available channels (pager, SMS, overhead).” “Assess ventilator-dependent patients’ vital signs and document on paper charts.” “Switch all charting to paper forms using the approved template.” “Contact Facilities to investigate the generator room per safety protocol.” “Reroute incoming patients to another hospital, notifying EMS and transport teams.”	**Persona Clarity:** Specifies “approved template” and “per safety protocol” to reinforce professional realism.
**Feedback Generation**	When the student types **End simulation and provide feedback**, deliver a structured debrief: **What they handled well** (e.g., adherence to evidence-based protocols, clear communication) **Critical issues they missed** (e.g., generator pre-check, multi-disciplinary coordination) **How to improve** (specific, actionable steps tied to guidelines and team-based communication) A 3–5-bullet summary or short paragraph with positive reinforcement and tailored improvement tips based on their experience level.	**Personalization):** Feedback tailored to experience level. **Credibility:** Links suggestions to guidelines. **Transparency:** Maintains clear, actionable format.
**Example Interaction Flow**	**Student:** I am the ER Charge Nurse (intermediate). **AI:** Power has failed across the hospital. Emergency lights are on; the generator has not kicked in. Three ventilator-dependent patients are critical. According to the hospital’s emergency checklist, what’s your first action? … **Student:** End simulation and provide feedback **AI:** Thank you. Here’s your feedback: You quickly prioritized ventilator checks per protocol—well done. Communication with ancillary staff was delayed; consider using the overhead paging system earlier. You followed generator-failure guidelines but missed a formal equipment inspection checklist. Next time, leverage your experience level to lead a brief team huddle before patient rerouting.	**Alignment:** Emergency Checklist. **Persona Clarity:** Contextualizes questions with guidelines. **Personalization:** Feedback reflects “intermediate” level.

## Data Availability

The original contributions presented in this study are included in the article/Supplementary Materials. Further inquiries can be directed to the corresponding author.

## Supplementary Materials

The following supporting information can be downloaded at: https://www.mdpi.com/article/10.3390/nursrep15080280/s1, File S1: Evaluation Framework Tools and Study Instruments; File S2: Instructional Manual: Applying the AIMS Evaluation Framework; File S3: Educational Chatbot Simulation Template

## Public Involvement Statement

No public involvement in any aspect of this research.

## Guidelines and Standards Statement

This manuscript was drafted in accordance with the GUIDED reporting guideline—*Guidance for Reporting Intervention Development Studies in Health Research* (O’Cathain A, Croot L, Duncan E et al. *BMJ Open.* 2019;9:e029954), because the primary aim was to develop and preliminarily apply a new educational evaluation framework (AIMS) for simulation-based nursing education. A completed GUIDED checklist is provided in the Supplementary Materials to demonstrate full compliance with each item.

## Use of Artificial Intelligence

Artificial intelligence (AI) and AI-assisted tools were used during this manuscript’s preparation to support multiple stages of development. Specifically, AI was used to refine early drafts of specific sections of the manuscript. The AI tool (ChatGPT-4, OpenAI) was also employed for language editing and improving grammar. Additionally, an AI-powered agent (“Eval-Bot”) was developed and trained on the dual-phase evaluation framework to simulate an assessment of a real chatbot prompt used in emergency response simulation. All AI-generated content was critically reviewed and revised by the authors to ensure accuracy, coherence, and compliance with scholarly standards.

## Abbreviations

The following abbreviations are used in this manuscript:

Abbreviation	Full term
AI	Artificial Intelligence
AIMS	AI-Integrated Method for Simulation Evaluation (dual-phase framework)
INACSL	International Nursing Association for Clinical Simulation and Learning
HSSOBP	Healthcare Simulation Standards of Best Practice^®^
ASPiH	Association for Simulated Practice in Healthcare
AACN	American Association of Colleges of Nursing
WHO	World Health Organization
FAITA	Framework for AI Tool Assessment in Mental Health
LLM	Large Language Model
SBE	Simulation-Based Education
CJM	Clinical Judgment Model
GPT-4	Generative Pre-trained Transformer 4 language model
ChatGPT	Chat Generative Pre-trained Transformer conversational agent
AB Bot	Assessment Bot—AI agent used to score prompts with AIMS
Eval-Bot	AI evaluator trained on AIMS rubric to automate scoring
3-bot	Three-Agent Validation Method (patient–provider–evaluator AI model)
