# Supplementary material for: Beyond the Bot: A Dual-Phase Framework for Evaluating AI Chatbot Simulations in Nursing Education
Source: Nurs Rep. 2025 Jul 31;15(8):280. doi: 10.3390/nursrep15080280 (PMC12389130; doi:10.3390/nursrep15080280)
Supplement: Supplementary file 1 [file nursrep-15-00280-s001.zip › Instructional Manual - AIMS.pdf]

Instructional Manual: Applying the AIMS Evaluation Framework

Overview

The AIMS Framework provides a structured method to evaluate AI-driven learning tools and prompts across six key domains. Each domain is scored on a 0–2 scale, then summed for an overall Phase 1 score (maximum 12). This manual guides you step by step.

---

1. Prepare for Evaluation

1. **Gather Materials**
   - The prompt, chatbot script, or learning activity to be evaluated.
   - AIMS domain definitions and scoring rubric (see Section 2).
2. **Assemble an Evaluation Team** (optional)
   - Ideally 2–3 subject-matter experts (clinical educators, instructional designers).
   - A facilitator to ensure consistency.

---

2. AIMS Domains & Scoring Rubric

Domain	Definition	Score 0	Score 1	Score 2
**Clinical/Content Credibility**	Use of evidence-based protocols, guidelines, or authoritative sources.	No reference to clinical guidelines or sources.	Vague or indirect references to best practices.	Explicit citation of current, reputable guidelines or protocols.
**Alignment**	Degree to which the tool's objectives, tasks, and complexity match the stated learning goals and learner level.	Learning goals unclear or misaligned with tasks.	Goals stated but only partially reflected in task design.	Clear, consistent mapping between goals, tasks, and learner complexity.
**Persona Clarity**	Transparency about the AI's identity, role, and responsibilities.	Bot identity or role is ambiguous.	Role is stated but may lack depth or contextualization.	AI's role, identity, and coach-vs-engine functions are clearly articulated.

Domain	Definition	Score 0	Score 1	Score 2
**Inclusivity & Cultural Competence**	Use of inclusive language, respect for diverse backgrounds, and consideration of cultural or accessibility needs.	No attention to inclusivity or cultural factors.	Some inclusive terminology or general statements (“we’ll be respectful”).	Proactive strategies (e.g., role-based language options, plain-language prompts, accessibility considerations).
**Transparency**	Clarity around AI’s non-human nature, purpose, and limitations.	Learner may be unsure they are interacting with AI or its scope.	AI identity is mentioned but without detail on purpose or limits.	Explicit, unambiguous disclosure of AI nature, scope, and how it will (and will not) support the learner.
**Personalization**	Customization to individual learners (e.g., role choice, experience level, proficiency).	No learner personalization.	Basic customization (e.g., role selection only).	Rich personalization—multiple learner attributes (role, experience, goals) guide content, pacing, and feedback.

---

3. Conducting the Evaluation

1. **Review the Tool**
    - Read through the entire prompt or simulation script.
    - Note any sections that reference protocols, learner instructions, persona statements, etc.
2. **Score Each Domain**
    - For each domain, assign 0, 1, or 2 based on the rubric.
    - Document your rationale in writing (to support consensus and improvement).
3. **Compute the Phase 1 Total**
    - Sum the six domain scores (max = 12).
4. **Identify Strengths & Gaps**
    - Highlight domains scoring 2 as strengths.
    - Domains scoring 0–1 indicate areas for revision.

---

4. Reporting Results

1. **Create an Evaluation Table**

**Domain**	**Score (0–2)**	**Rationale**
Clinical/Content Credibility		
Alignment		
Persona Clarity		
Inclusivity & Cultural Competence		
Transparency		
Personalization		
**Total Phase 1 Score**	** / 12**	

2. **Write a Summary Narrative**
   - 1–2 paragraphs summarizing overall performance, key strengths, and prioritized recommendations.
3. **Recommend Next Steps**
   - For each low-scoring domain, suggest concrete revisions (e.g., “Add explicit citation of XYZ guideline” or “Include learner experience-level input”).

---

5. Example Application

Clinical/Content Credibility (Score 0):
The prompt contains no reference to any emergency management protocol.
Recommendation: Cite the latest AHA “Code Blue” guidelines to ground clinical realism.

Repeat for each domain, then compile the total and narrative.

---

6. Iterative Revision & Re-evaluation

- After updates, rerun the AIMS evaluation to verify improvements.
- Aim to reach at least 10/12 for a robust, learner-centered AI simulation.

End of Manual
Use this guide to ensure your AI simulations and prompts meet rigorous standards for educational efficacy, credibility, and inclusivity.
