# Supplementary material for: Beyond the Bot: A Dual-Phase Framework for Evaluating AI Chatbot Simulations in Nursing Education
Source: Nurs Rep. 2025 Jul 31;15(8):280. doi: 10.3390/nursrep15080280 (PMC12389130; doi:10.3390/nursrep15080280)
Supplement: Supplementary file 1 [file nursrep-15-00280-s001.zip › Supplementary_File_1_Evaluation_Framework (1).pdf]

Supplementary File 1: Evaluation Framework Tools and Study Instruments

S1. Eval-Bot Prompt and Description

Bot Name: Eval-Bot

Purpose: To evaluate chatbot prompts using the Phase 1 evaluation criteria of the Dual-Phase Framework and provide structured feedback.

Prompt to Create Eval-Bot:

You are Eval-Bot, an AI assistant trained to assess educational chatbot prompts using the Dual-Phase Evaluation Framework.
You will score the prompt across seven domains: Content Credibility, Educational Alignment (Instructional & Cognitive),
Safety and Risk Mitigation, Inclusivity and Cultural Competence, Transparency, and Personalization.
For each domain, assign a score from 0 (Not Addressed) to 2 (Fully Addressed), provide a brief rationale, and suggest improvements if needed.

Usage Note: Eval-Bot was used to evaluate the Emergency Response Simulation Bot prompt as described in the manuscript.

S2. Phase 2 Evaluation Design: Measuring the Educational Impact of the Chatbot Simulation

Objective

To evaluate how the AI chatbot simulation affects students’ learning outcomes, critical thinking, decision-making, and overall user experience in emergency response training.

Methodology

1. Mixed-Methods Approach
This evaluation will combine **quantitative surveys** and **qualitative reflections** to assess both the measurable and experiential aspects of student engagement with the chatbot.

Quantitative Component

Instrument:
A Likert-scale survey developed using validated educational technology and simulation frameworks (e.g., Technology Acceptance Model, INACSL standards).

Sample Survey Items (Likert Scale: Strongly Disagree [1] – Strongly Agree [5]):

Domain	Survey Item
**Content Accuracy**	The chatbot provided clinically accurate and relevant information.
**User Experience**	I found the simulation easy to navigate and engaging.
**Dialogue Control**	I felt I could influence the direction of the conversation effectively.
**Learning Outcomes**	The simulation improved my ability to make decisions in emergency scenarios.
**Feedback and Assessment**	The chatbot gave useful feedback based on my responses.
**Equity & Accessibility**	The simulation content reflected diverse patient populations.
**Transparency**	It was clear that I was interacting with an AI and not a real person.
**Interactivity Quality**	The chatbot's responses felt realistic and kept me engaged.

Data Analysis:
Descriptive statistics, t-tests, and ANOVA may be used to compare differences across groups (e.g., role selected, prior simulation experience).

Qualitative Component

Data Collection Methods:

- Open-ended survey questions (e.g., "Describe how the chatbot supported or hindered your learning.")

- Reflective journaling prompts
- Optional focus groups or interviews

Key Themes for Coding:

- Clinical reasoning and critical thinking
- Trust and believability of chatbot
- Cultural responsiveness
- Perceived realism and emotional engagement

Analytical Approach:
Thematic analysis using NVivo or similar qualitative software, with coding mapped to the eight Phase 2 domains.

Outcomes Measured

- Improvement in decision-making and scenario-based problem-solving
- Learner satisfaction and trust in AI tools
- Effectiveness of AI feedback for formative learning
- Identification of domain-specific performance gaps (e.g., inclusivity or interactivity)
